# Instrumentalized 3D printed scaffolds enable bone regeneration and fracture healing monitoring in critical size bone defects

**DOI:** 10.1063/5.0295886

**Published:** 2026-08-21

**Authors:** Gerardo Figueroa Romero, David A. Gonzales, Ginelle Maldonado, Stephany R. Maldonado, Joselyn M. Toothaker, Annemarie E. Rau, Efren Barron Villalobos, Robert V. Childers, Sabina M. Romero, Paige E. Rudy, Gayatri Kaimal, John A. Szivek, David S. Margolis

**Affiliations:** 1 Department of Orthopedic Surgery, University of Arizona, Tucson, Arizona 85721, USA; 2 Department of Biomedical Engineering, University of Arizona, Tucson, Arizona 85721, USA; 3 Department of Physiological Sciences, Bio5 Institute, University of Arizona, Tucson, Arizona 85721, USA; 4 Department of Orthopaedic Surgery, Medical College of Wisconsin, Milwaukee, Wisconsin 53226, USA

## Abstract

Critical size bone defects do not heal without surgical intervention, and the current methods of treatment lack long-term efficacy and patient satisfaction. Our lab has previously demonstrated the use of 3D printed scaffold mimicking biological tissue structure in the regeneration of large critical size bone defects. In this study, we highlight the effect of dynamization and hardware removal in the regeneration of critical size bone defects treated with our instrumentalized 3D printed polymer scaffold. We demonstrate the sensate capabilities of our scaffolds in long-term monitoring of bone healing and bone-implant biomechanics. All scaffold-implanted groups demonstrated rapid bone growth in the defect space. Dynamization resulted in an increased union rate and bone volume. The hardware removal group demonstrated increased bone stiffness compared to the non-dynamized and control groups.

## INTRODUCTION

Critical size mid-diaphyseal bone defects can occur following high energy trauma, bone cancer resection, or disease.^1^ Current treatment options include bone allografts and autografts, bone transport, and vascularized grafts, but none of them offers consistently high levels of safety, patient satisfaction, and long-lasting efficacy.^2^ The treatment of mid-diaphyseal bone defects in weight-bearing long bones possesses an additional risk due to high rates of nonunions, as high as 20%.^3^

3D printed scaffolds are an emerging approach for treating bone defects. The adoption of high-resolution imaging capabilities such as micro-computed tomography (*μ*CT) has resulted in the development of tailored scaffolds with a biological structure patterned on the patient's tissues.^4–6^ Furthermore, the addition of progenitor cells to bioactive scaffold surface coatings facilitates bone regeneration.^7,8^

Our lab has previously focused on using polybutylene terephthalate (PBT) for tissue engineering applications in bone tissue. Early studies demonstrated extensive bone growth with pure PBT scaffolds.^9^ The addition of β-tricalcium phosphate (TCP) particles on PBT scaffold surfaces provided an osteoconductive layer for osteogenic differentiation *in vitro*^10^ and higher bone growth *in vivo.*^11–13^ Bone growth can further be enhanced through the use of a biomimetic design, allowing for over 500% more bone ingrowth in a canine model.^14^ In a pilot study, pure PBT scaffolds alone were not sufficient for healing a critical size bone defect in a sheep model, but the addition of endogenous adipose derived stem cells (ASCs) allowed for complete union across the defect space in 6 months.^15^

Fracture healing is a complex process. Biological and mechanical cues in the fracture site's microenvironment dictate the differentiation of progenitor cells and the osteogenic activity of osteoblasts responsible for bone formation.^16^ Stiff fixation devices limit interfragmentary movement, often leading to reduced bone formation and delayed healing.^17^ Dynamization, performed as the removal of one or more interlocking screws in intramedullary nails, has been shown to increase interfragmentary loading and strain, stimulating osteogenesis.^18–20^ Other secondary surgeries, such as implant removal, increase bone loading and have demonstrated further bone healing several months post-surgery.^21^

One concern of secondary surgery to dynamize or remove an implant is the risk of refracture due to instability.^22,23^ The gold standard for monitoring bone healing is through radiographic imaging. Radiographs cannot provide real-time quantitative assessment of bone healing progression and expose the patient to radiation.^24^ The incorporation of wireless electronic devices to fixation implants and scaffolds, providing real-time measurements, has become a growing field to better understand fracture healing and its microenvironment.^25,26^ Our lab has previously utilized strain gauges attached to 3D printed polymer scaffolds for real-time assessment of the mechanical environment within focal and large hyaline cartilage defects.^12,13^ These studies highlighted the potential of this technology referred to as “sensate,” to advance the assessment and monitoring of musculoskeletal tissues and healing.

In this study, we highlight the effect of dynamization and hardware removal in the regeneration of large critical size bone defects using a biomimetic 3D printed ceramic-coated polymer “sensate” scaffold. The study was focused on the use of our sensate capabilities for the characterization and monitoring of implant-site biomechanics and bone regeneration, respectively.

## RESULTS

### Scaffold characterization

**FIG. 1.**
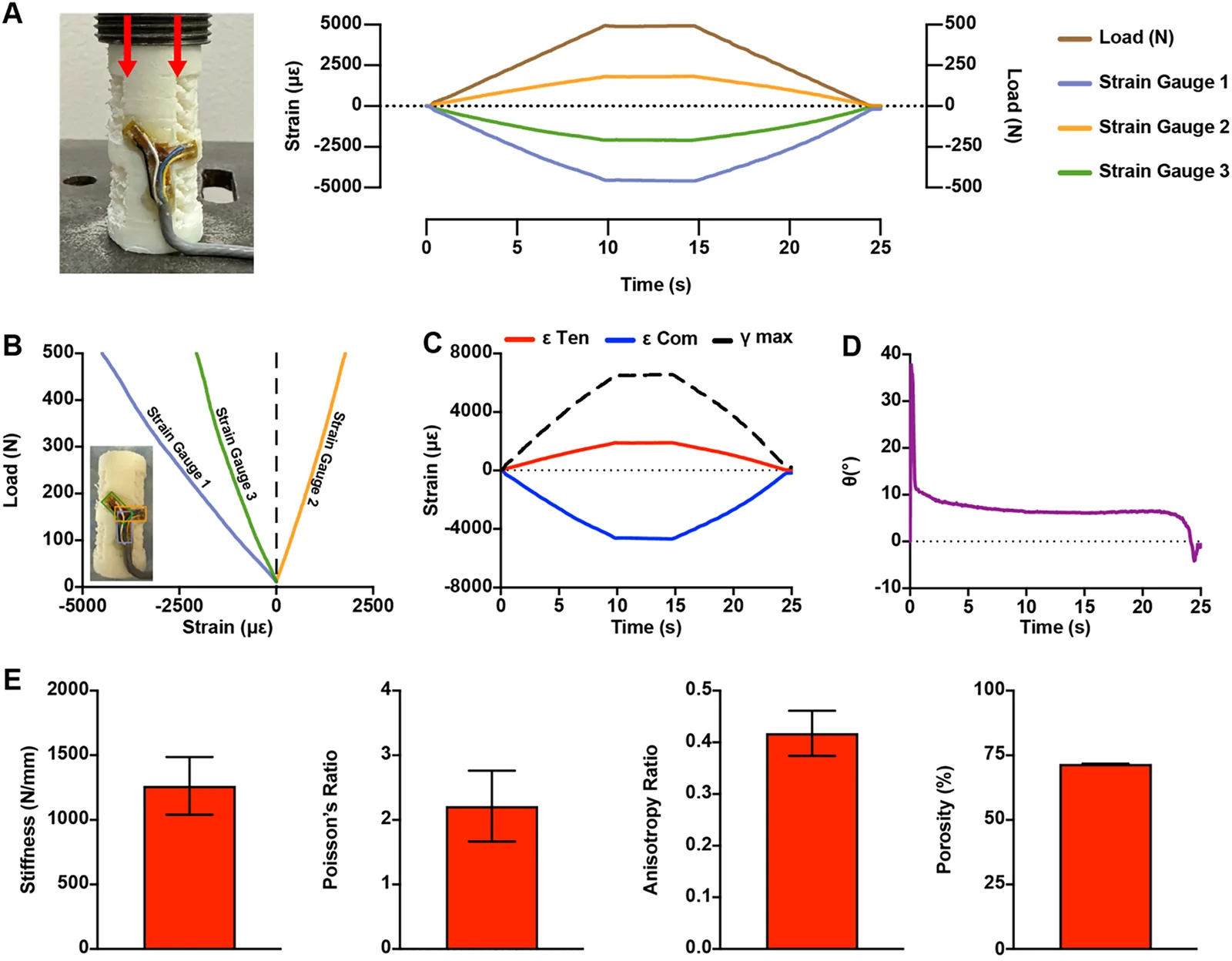
Mechanical and structural characterization of 3D printed biomimetic scaffolds. (a) Load and strain curves obtained from compressive mechanical testing. Strain gauges 1 (blue), 2 (orange), and 3 (green) showing a linear relation to load changes (brown). (b) Load v. Strain curves of individual scaffolds under compressive testing, the slope was used to calibrate each individual scaffold. (c) Graphical representation of the principal strains (Red: Tensile principal strain, Blue: Compressive principal strain, Dashed: maximum shear strain). (d) Change in principal angle, recorded under compressive loading. (e) Mechanical and structural characterization of porous biomimetic scaffolds. Data are shown as the mean ± standard deviation.

The biomimetic pattern inside the cylindrical scaffolds resulted in an overall porosity of 71.6 ± 0.2% [Fig. 1(e)]. Nondestructive mechanical testing demonstrated a linear elastic relation in the load vs strain curves up to 498 N [Figs. 1(a) and 1(b)]. The principal strains and maximum shear strain showed a similar linear elastic response during mechanical loading [Fig. 1(c)]. During the initial loading phase, the principal angle showed a spike, reaching 33° and then steadily dropped to 6° for most of the loading cycle [Fig. 1(d)]. Scaffolds had an average stiffness of 1262 ± 223.3 N mm^−1^ with a Poisson's ratio of 2.21 ± 0.55. Scaffolds showed a high degree of anisotropy during mechanical testing with an average ratio of 0.41 ± 0.04 [Fig. 1(e)].

### Cell isolation and seeding

In supplementary material, Fig. 5, we demonstrate the ability of the autologous derived stem cells to attach to the scaffold surface before implantation. Segmentation of the cells on the *μ*CT images demonstrated an even distribution of attached cells throughout the scaffold.

### *In vivo* sensing measurements

Limited data were obtained from the implanted telemetry system due to waterproofing issues, and for this reason, most animals in the study had wired devices. In animals with working telemetry systems, sample strain data are shown in supplementary material, Fig. 6.

**FIG. 2.**
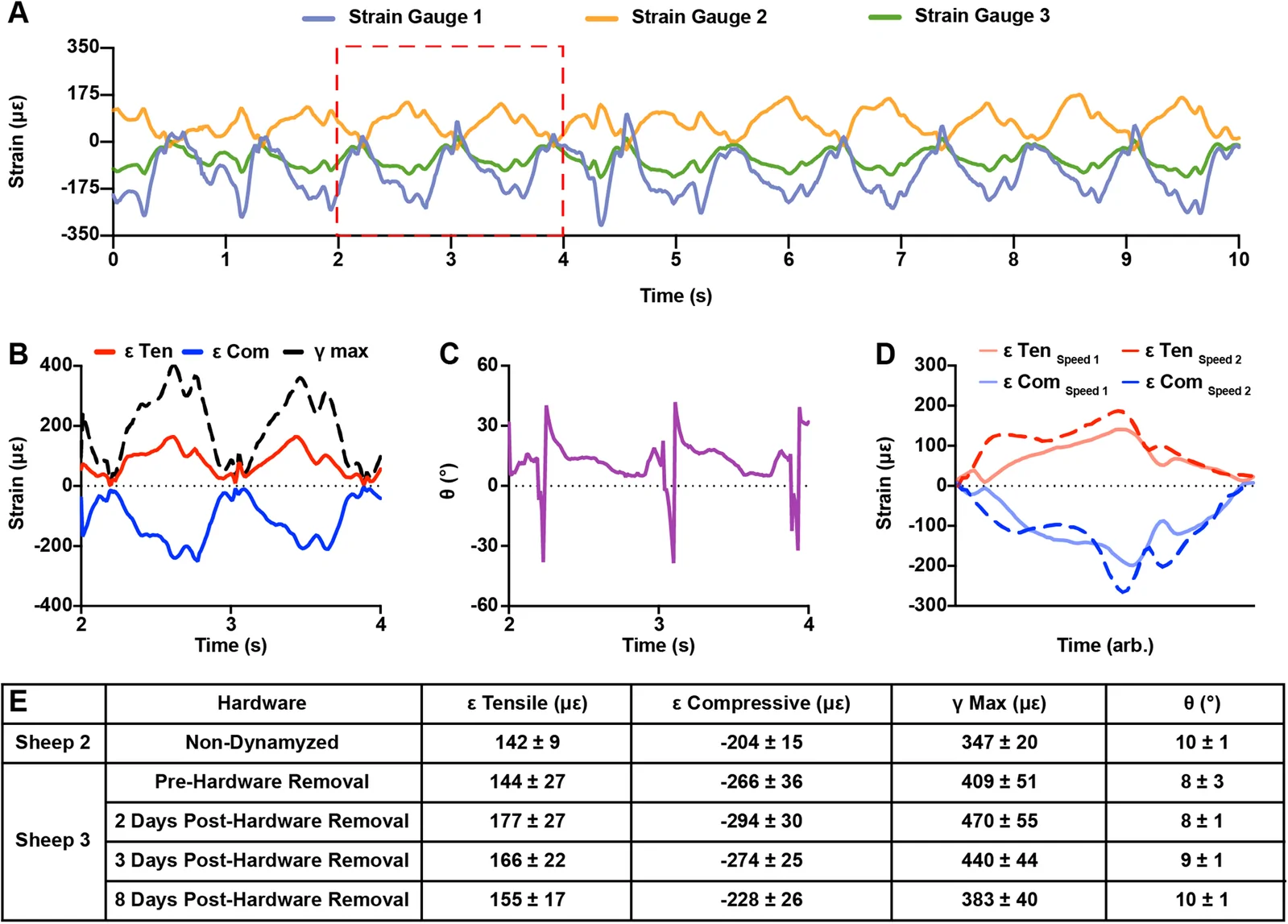
Principal strains in implanted scaffolds during gait. (a) Representative strain data from the custom rosette strain gauge during treadmill walking. The segment highlighted by the red dashed square depicts 2 s of walking, corresponding to 2 gait cycles. Raw data are used to observe ε Tensile (b, red), ε Compressive (b, blue), γ Max (b, dashed), and θ (c). (d) Tensile and compressive principal strains of an animal treadmill walking at two different speeds. (e) Changes in average ε Tensile, ε Compressive, γ Max, and θ of implanted scaffolds in a non-dynamized and hardware removal sheep. Values were assessed at the point of highest compressive and tensile strains, just prior to lift-off in the experimental limb. Data are shown as mean ± standard deviation.

Sample strain data from rosette components are shown in Fig. 2(a). All gauges with similar orientation showed similar strain patterns during treadmill walking. Video and strain data synchronization showed 4 distinct phases of gait: lift-off, mid-swing, touch-down, and mid-stance (supplementary material, Fig. 7). During walking, before the test limb leaves the ground, the scaffold is at the point of maximum compression along its longitudinal axis (1). During swing phase, the scaffold undergoes tension (2) until touch-down (3). Rapid compression follows when the contralateral limb leaves the ground, and the animal begins mid-stance on the test limb (4).

Graphical representations of the principal strains, the maximum shear strain, and the principal angle during gait are shown in Figs. 2(b) and 2(c). During the beginning of mid-stance, the tensile and compressive principal strains, and the maximum shear strain are at their lowest magnitude. At this time, there is a large change in the principal angle from −38° to 40°. As both principal strains and maximum shear strains increase in magnitude, the principal angle decreases until it reaches a plateau around 13°. The principal strains and maximum shear strain reach their highest magnitude at the end of mid-stance before decreasing when the animal begins the swing phase.

Supplementary material, Fig. 8 illustrates the changes in principal strain magnitudes on the scaffold surface during gait. The principal strain angle is represented as the angular change between the principal tensile strain and the scaffold width, with the principal compressive strain acting at 90° to the axis along the scaffold width. As the limb approaches touch-down, the compressive principal strain axis begins to align along the long-axis of the scaffold. Between touch-down and the beginning of mid-stance, the scaffold is under tension before returning to a compression state at the end of mid-stance. Before the beginning of lift-off and mid-swing, the scaffold primarily experiences compressive deformation, which is nearly aligned with the long axis of the scaffold.

Walking at higher speed increased tensile, compressive, and maximum shear strains in both non-dynamized (Sheep 2) and dynamized (Sheep 3) animals [Fig. 2(d) and supplementary material, Fig. 9]. In non-dynamized animals, the compressive principal strains showed the highest increase in magnitude (−204 ± 15 to −266 ± 14 *μ*ε) compared to the dynamized animals (−266 ± 36 to −286 ± 16 *μ*ε). Both groups demonstrated a decrease in the principal angle while walking at higher speeds.

Principal strain calculations, taken at the maximum magnitude of tensile and compressive strains (before lift-off), are presented for two sheep: one non-dynamized (Sheep 2) and one that underwent hardware removal (Sheep 3). The non-dynamized animal showed an average principal tensile strain of 142 ± 9 *μ*ε, a principal compressive strain of -204 ± 15 *μ*ε, and a maximum shear strain of 347 ± 20 *μ*ε. Strain data from Sheep 3, which underwent hardware removal, demonstrated an increase in principle tensile strain (144 ± 27 to 177 ± 27 *μ*ε), principal compressive strain (−266 ± 36 to −294 ± 30 *μ*ε), and maximum shear strain (409 ± 51 to 470 ± 55 *μ*ε) between pre-removal and 2 days following hardware removal. Both principal strains and maximum shear strains decreased between 2- and 8-days post-surgery, while the principal angle increased.

Strain results using single axis measurements are presented for 6 sheep (supplementary material, Fig. 10). Comparisons across time were only made for strains from the same gauge.

**FIG. 3.**
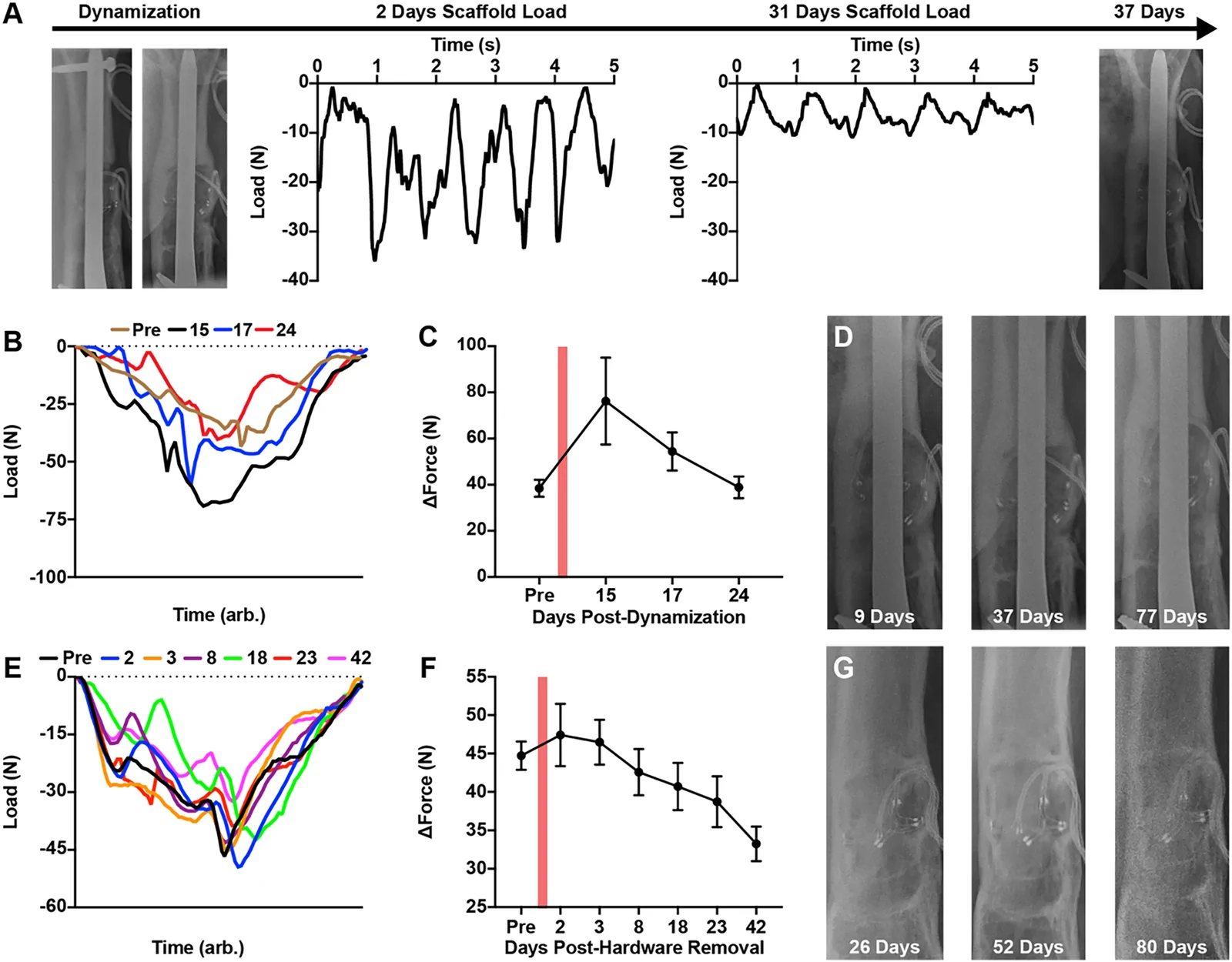
Real-time monitoring of scaffold loads recorded from implanted sensors during treadmill walking following dynamization and hardware removal. (a) Illustration for the use of scaffold loads as an assessment for bone healing. Following dynamization, scaffold loads decreased between 2 and 31 days correlating with radiographic changes at 37 days. (b) Sample scaffold loading cycles and (c) changes in scaffold loads 15-, 17-, and 24-days post-dynamization. (d) Representative radiographic images of a sheep 9-, 37-, and 77-days following dynamization. (e) Sample scaffold loading cycles and (f) changes in scaffold loads of a sheep 2-, 3-, 8-, 18-, 23-, and 42-days post-hardware removal. (g) Representative radiographic images of a sheep 26-, 52-, and 80-days following hardware removal. Data in (c) and (f) are shown as mean ± standard deviation with the red line representing the secondary surgery.

Figure 3(a) demonstrates the use of scaffold loads to assess bone healing. Following dynamization, a clear increase in loads on scaffolds occurred between 2- and 31-days post-surgery. Three animals demonstrated similar changes in scaffold loads post-dynamization (supplementary material, Fig. 10. **Sheep 4, 5, and 6**). Representative changes in force across the scaffold during gait are depicted in Figs. 3(b) and 3(c) prior to and following dynamization. Force passing through the scaffold increased 100% after 15 days following dynamization and then decreased 48.68% by day 24. Sheep #5 showed the highest decrease in load of 68.97% between days 2 and 31.

A change in scaffold forces was observed in an animal undergoing hardware removal (Sheep #3) [Figs. 3(e) and 3(f)]. Following surgery, that animal experienced a 6% increase (45 ± 2 to 47 ± 4 N) in compressive forces, decreasing 29.88% by day 42 (33 ± 3 N).

### Radiographs and mechanical testing

**FIG. 4.**
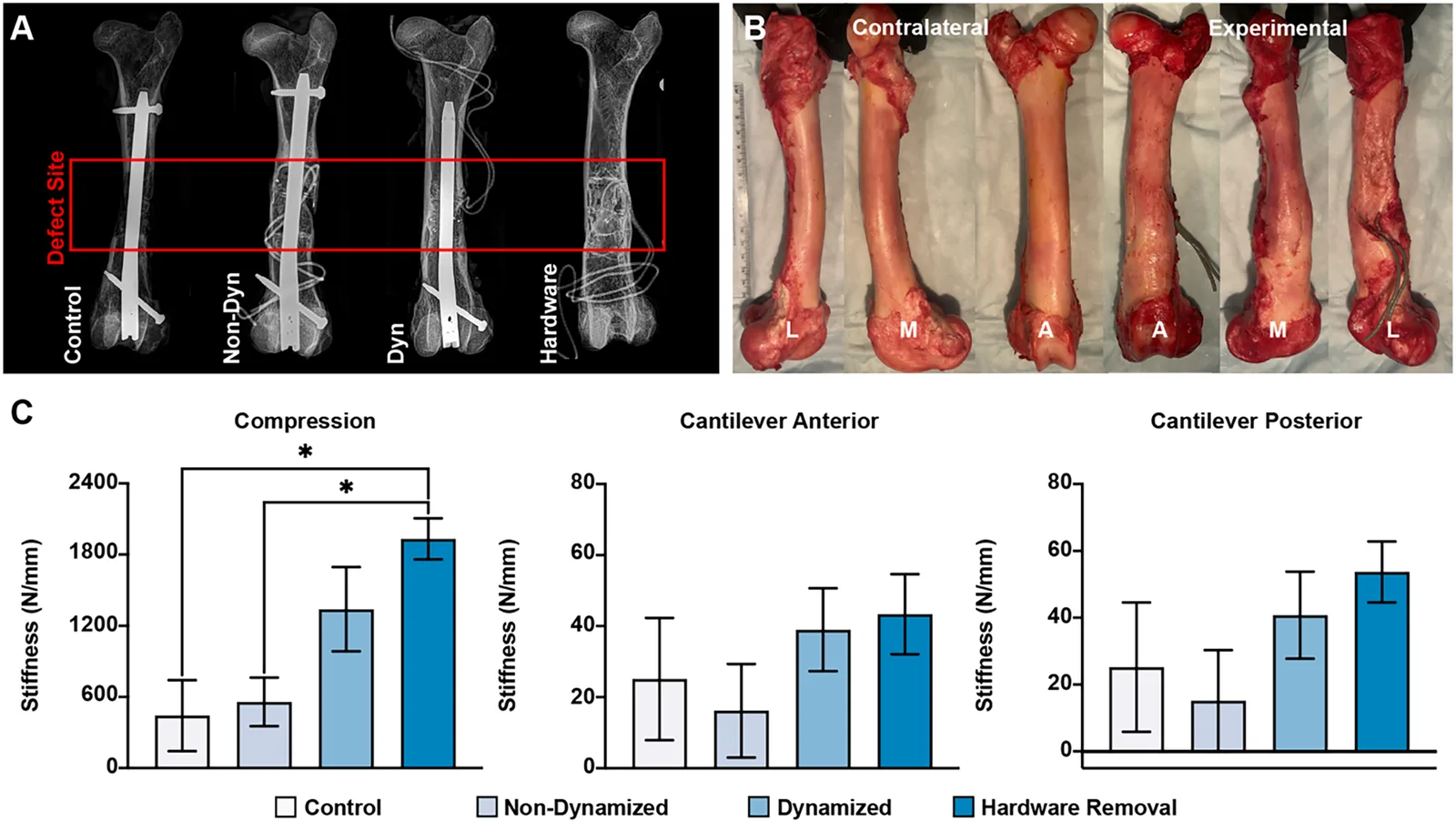
Explantation and mechanical testing of experimental femora. (a) Anterior radiographic images of explanted experimental femora highlighting the defect space. (b) Representative images of a hardware removal sheep femora following euthanasia. The anterior (A), medial (M), and lateral (L) views of the intact (contralateral) and experimental femurs are shown. (c) Mechanical testing results for mechanical testing including axial compression, cantilever anterior, and cantilever posterior testing. Data for all bar graphs are shown as mean ± standard error of the mean with statistical significance at ^*^p < 0.05.

Following 9 months *in vivo*, radiographs demonstrated union in one of three control sheep. Two of six sheep in the non-dynamized group, five of six sheep in the dynamized group, and four of four sheep in the hardware removal group showed radiographic union. Representative radiographic images for control (I), non-dynamized (II), dynamized (III), and hardware removed (IV) groups are shown in Fig. 4(a). Gross examination of explanted femora demonstrated bone growth in all groups with scaffolds [Fig. 4(b)].

One femur from a control and one from a dynamized sheep failed during preparation for mechanical testing and were reported in the stiffness analysis as 0 N/mm. Four additional bones from the non-dynamized group failed during testing, and their stiffness was reported as 0 N/mm. Axial compressive testing demonstrated a significant bone stiffness increase in the hardware removed group (1932 ± 346.7 N mm^−1^) compared to the control (442.3 ± 518.5 N mm^−1^) and non-dynamized (558.1 ± 500.5 N mm^−1^) groups (*p* < 0.05) [Fig. 4(c)]. When normalizing whole bone stiffness of the regenerated bone to the contralateral intact femora, the hardware removal group (141.5 ± 72.53%) demonstrated significantly higher stiffness than the control group (17.09 ± 19.80%) (p < 0.05) (supplementary material, Fig. 11). The hardware removal group showed a trend toward increased stiffness in anterior and posterior cantilever bending that was not statistically significant (*p* > 0.05).

### *μ*CT

**FIG. 5.**
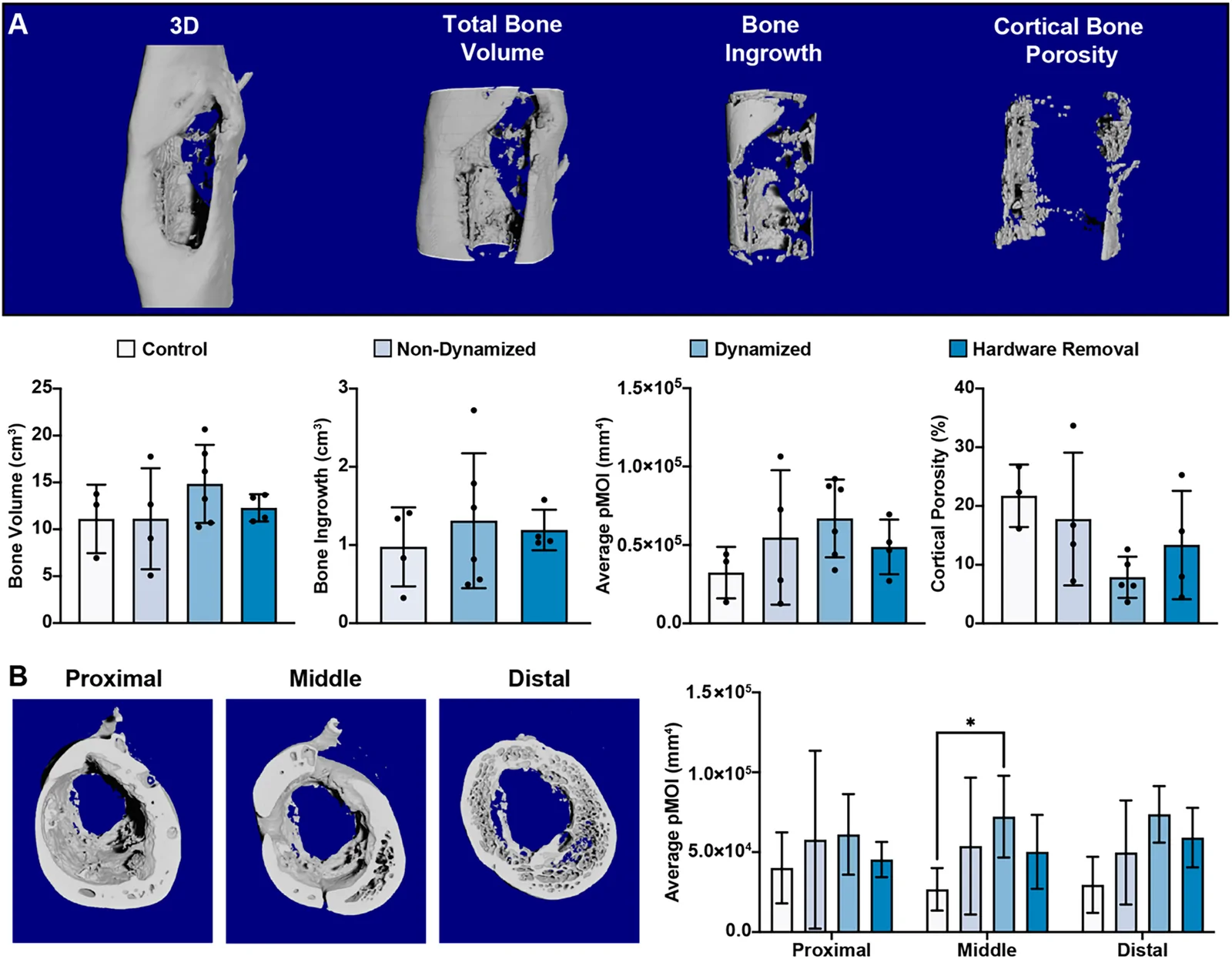
*μ*CT imaging analysis of regenerated bone segments. (a) (Top) 3D reconstruction of the femoral diaphysis and representative images of the parameters obtained. (Bottom) Quantitative μCT parameters including bone volume, bone ingrowth, average pMOI, and cortical porosity. (b) Assessment of pMOI along three sites in the defect space, proximal, middle, and distal. Data for all bar graphs are shown as mean ± standard deviation with statistical significance at ^*^p < 0.05.

Quantitative *μ*CT measurements are highlighted in Fig. 5. Femora from the dynamized sheep (14.85 ± 4.16 cm^3^) showed a higher cortical bone volume compared to controls (11.11 ± 3.65 cm^3^) and non-dynamized (11.13 ± 5.39 cm^3^) groups (p > 0.05). There was a trend toward decreasing bone volume in the hardware removal group (12.29 ± 1.44 cm^3^) (p > 0.05). Non-dynamized bones showed the lowest bone ingrowth into the scaffold pores (0.98 ± 0.51 cm^3^). Ingrowth increased following dynamization (1.31 ± 0.86 cm^3^) and decreased following hardware removal (1.19 ± 0.26 cm^3^) (p > 0.05). Dynamized femora demonstrated the highest average pMOI (66 929 ± 24 838 mm^4^) compared to other groups (control: 32 371 ± 16 433 mm^4^, non-dynamized: 54 814 ± 42 842 mm^4^, hardware removal: 48 775 ± 17 442 mm^4^). Cortical bone porosity progressively decreases between control, non-dynamized, and dynamized femora (21.74 ± 5.31%, 17.78 ± 11.31%, and 7.86 ± 3.50%, respectively) and increased in the hardware removed group (13.36 ± 9.24%). The pMOI at the three defect regions (proximal, middle, and distal) showed a similar trend to the overall average pMOI across the whole defect space [Fig. 5(b)]. In the middle of the defect space, the dynamized femora (72 215 ± 25 596 mm^4^) demonstrated a significantly higher pMOI than the control femora (26 741 ± 13 359 mm^4^) (*p* < 0.05).

### Histology

**FIG. 6.**
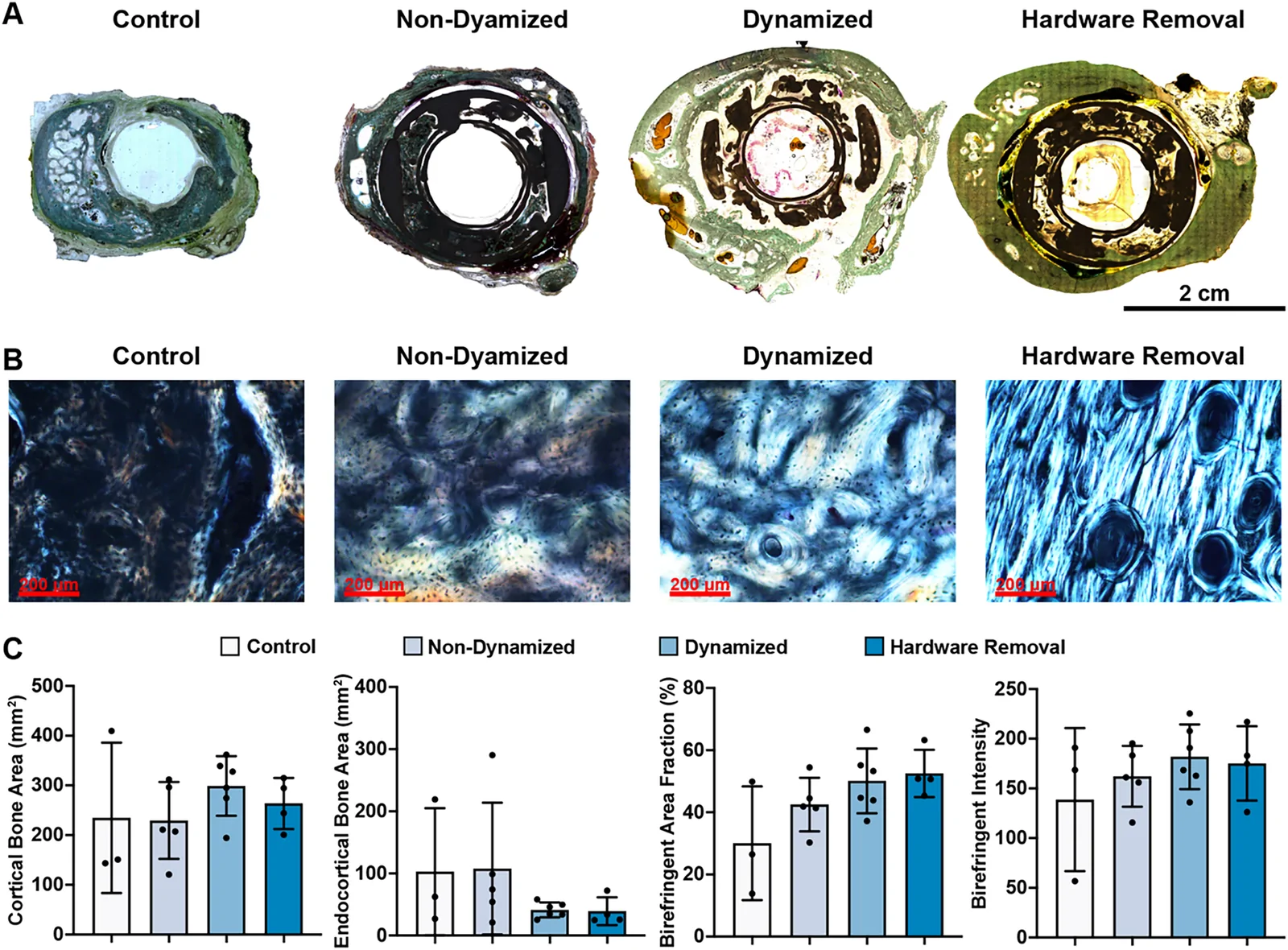
Histological evaluation of regenerated femora under brightfield and polarized light microscopy. (a) Representative brightfield images of mid-diaphyseal cuts of the regenerated bone segment in control, non-dynamized, dynamized, and hardware removal animals. (b) Representative polarized light microscopy images of regenerated femora of control, experimental groups, and intact contralateral bone. (c) Quantitative histological analysis of bone segments under brightfield and polarized light microscopy, including cortical bone area, endocortical bone area, birefringent area fraction, and birefringent intensity. Data for all bar graphs are shown as mean ± standard deviation.

Histological analysis is shown in Fig. 6 including representative brightfield [Fig. 6(a)] and polarized light microscopy [Fig. 6(b)] images as well as quantitative histological analysis [Fig. 6(c)]. Dynamized femora demonstrated the highest cortical bone area (298.7 ± 59.91 mm^2^) when compared to control (234.5 ± 151.4 mm^2^), non-dynamized (229.4 ± 77.37 mm^2^), and hardware removal groups (263.5 ± 51.41 mm^2^) (p > 0.05). Endocortical bone area progressively decreased across the control, non-dynamized, dynamized, and hardware removal groups (102.8 ± 102.2 mm^2^, 107.7 ± 106.1 mm^2^, 41.28 ± 12.02 mm^2^, and 39.09 ± 22.41 mm^2^, respectively) (p > 0.05). Polarized light microscopy demonstrated a trend toward increasing birefringent area across the control, non-dynamized, dynamized, and hardware removal groups. Birefringent intensity was similar in the dynamized and hardware removal groups.

## DISCUSSION

Our results demonstrated that the dynamization of large critical size femoral defects treated with autologous stem cell seeded TCP coated PBT scaffolds regenerate functional bone. This is highlighted by the ability of regenerated long bones to withstand physiologic load bearing in a large animal model following hardware removal. Radiographs, mechanical testing, *μ*CT scans, and histology confirmed changes in bone parameters in dynamized bones and following hardware removal compared to non-dynamized femora.

TCP coated biomimetic PBT scaffolds have previously demonstrated accelerated bone growth in small animal models and bone bridging across a 4.2 cm defect when seeded with autologous adipose derived stem cells.^9,14,15^ This study did not test whether the use of adipose derived stem cells contributed to healing the large critical size defects as all scaffolds were seeded with autologous cells 1 day prior to surgery. Prior studies from our lab have shown that adipose derived stem cells remain viable for up to 2 weeks in cell culture on PBT scaffolds, and that cells secrete alkaline phosphatase, indicating osteogenic activity.^30^ This paper demonstrated that gold nanoparticle infused autologous adipose derived stem cells were evenly distributed throughout the scaffold. One limitation of this, and our prior study, is that assays were not performed to confirm trilineage differentiation potential of the autologous stem cells into osteoblastic, chondrogenic, and adipogenic phenotypes.

Utilizing custom made rosette strain gauges, we quantified the mechanical and structural characteristics of scaffolds, which had a porosity of 71.60 ± 0.20%. Several studies have emphasized the importance of porosity in scaffolds to facilitate cell migration, vascularization, and osteogenesis.^31–33^ Jiao *et al.* demonstrated that trabecular tantralum with a 70% porosity resulted in better osteogenic differentiation, bone ingrowth, and osteointegration compared to scaffolds with 60 or 80% porosity.^34^ The mean scaffold stiffness was 1262 ± 223 N/mm, exhibiting a linear load vs strain relationship during calibration, as previously observed.^11,12,35,36^ Although our measured scaffold stiffness is higher than other scaffolds used in weight-bearing bone defects,^37^ a higher stiffness scaffold can be beneficial in cell migration and stem cell differentiation in the reconstruction of cortical bone defects.^38^

To increase osteogenesis in our study, we dynamized intramedullary nails at 6 months following implantation. Clinically, nail dynamization has been demonstrated to improve union rates of long-bone fractures.^18,39^ In contrast to other study designs in which dynamization was carried out in earlier stages of fracture healing, we performed the procedure 6 months following implantation. Early dynamization or exposure to less stiff fixation often results in a more callus formation but with less mineralization.^40–42^ Based on our pilot study, at 6 months, a large portion of the defect space is filled by bony callus, making it optimal for dynamization. The modified intramedullary nail used in this study has been shown to provide excellent fixation in a sheep model, limiting interfragmentary movement and securing the scaffold until bone growth occurs in the defect space.^15,28^ Femora in the dynamized group demonstrated both increased union rates and bone stiffness in axial compression, cantilever anterior, and cantilever posterior bending when compared to non-dynamized femora and controls. *μ*CT demonstrated that dynamized femora showed the highest cortical bone volume, bone ingrowth, pMOI, and lowest cortical bone porosity. Similar results were observed in quantitative histological evaluation, with dynamized femora having the highest cortical bone area. A similar increase in mechanical properties has been noted following dynamization of long-bone fractures of both small and large animals.^19,20,43^ In contrast to other studies describing a lower callus size in delayed dynamization compared to static fixation,^20^ we believe our increased bone volume and bone ingrowth signify that dynamization at 6 months still encouraged additional bone formation in the defect space.

Another limitation to this study is that a control group using current surgical methods to address bone loss was not included. Methods include the use of bone allograft and autograft, bone transport, and endoprosthesis.^44^ Each method has potential drawbacks. For example, autograft has limited availability, causes donor site morbidity, and, in the case of vascularized grafts, requires a surgeon skilled in free tissue transfer.^45^ Allograft has the potential for disease transmission and immune rejection as well as limited osteoinductive potential compared to autograft.^44,46^ Although our study did not directly compare PBT scaffolds to these methods, the healing potential of our scaffolds warrants additional investigation as an off-the-shelf product that could be customized to an individual patient and can provide another surgical strategy for healing large critical size defects and may be indicated in cases where the use of autograft or allograft fails. Cell seeding density and time between seeding cells on a PBT scaffold and surgery should be studied as these may significantly alter the regenerative potential of this treatment strategy but were beyond the scope of the current study.

Clinically, complete hardware removal is indicated when there is implant failure, infection, or pain.^47^ In patients undergoing implant removal, a decrease in pain scores with improved functional outcomes was observed.^48,49^ In a study by Lee *et al.*, an increase in mean gap-filling rate was seen 2 years after implant removal, signifying continuous bone healing progression.^21^ In our study, we conducted a complete hardware removal in 4 sheep that had undergone dynamization. In the hardware removed group, we observed that femora consistently had the highest stiffness in compressive axial, cantilever anterior, and cantilever posterior loading. *μ*CT revealed a decrease in bone volume, bone ingrowth, and average pMOI compared to dynamized femora. Histological evaluation demonstrated a similar trend in cortical bone area. The decrease in *μ*CT and histologically measured parameters relative to dynamized femora can be explained by active remodeling in the regenerated bone. As explained by Wolff's law,^50^ bone remodeling will occur based on the mechanical environment experienced by the bone. Following hardware removal, femora experience normal physiological loads, encouraging remodeling through callus resorption. Polarized light microscopy highlighted the change in the birefringent area fraction, percent of space occupied by organized collagen, demonstrating an increasing trend toward the hardware removal group. These results highlighted that performing hardware removal results in a higher percent of organized collagen and increased mechanical properties, consistent with having a more organized bone matrix.^51^

In this study, hardware removal was performed to demonstrate that the regenerated bone could withstand physiologic loading for an extended period without supporting hardware and was not designed to test or support asymptomatic hardware removal in patients. It is also important to note that the *ex vivo* analysis of bones from the non-dynamized and dynamized sheep occurred at the same time point following the creation of the critical size defect, but the *ex vivo* analysis of bones from the hardware removal sheep occurred later. It is possible that bones from sheep in the non-dynamized and dynamized groups would have demonstrated improved stiffness and increased woven bone at a later time. For this reason, the data presented in this study do not provide evidence that hardware removal is beneficial and should only be interpreted to mean that bone defects regenerated using the strategy outlined in this paper are able to withstand physiologic loading without fracture.

One concern with secondary surgeries including dynamization or hardware removal is the potential of refracture^23,52^ due to the presence of stress risers. To better understand healing dynamics and appropriately perform secondary surgeries, methods of assessing bone healing progression and mechanics are needed. Previous studies have focused on the technological development of sensate fixation implants and scaffolds for monitoring physiological loads as well as local disease progression. In the treatment of bone defects, strain gauged fixation plates have been utilized for implant load monitoring during healing.^53–55^ Windolf *et al.*^53^ reported the use of a smart plate for monitoring a tibial defect with a 30 mm gap. They observed a 50% reduction in implant loads by day 234. Fountain *et al.* demonstrated the utility of a smart implant in bone tissue engineering models.^54^ In sheep with a 30 mm defect treated with a polycaprolactone scaffold, the smart implant was accompanied by a body harness containing an accelerometer. They demonstrated that a smart implant in combination with wearable technology provided animal activity and loading events, but no trends were noted to support its use in monitoring long-term fracture healing. To our knowledge, this is the first report of an instrumented scaffold being used in the treatment of critical bone defects for continuous load monitoring at the fracture site to measure biomechanics and healing.

Synchronization of video and strain data demonstrating scaffold deformations in different phases of gait showed similar strain patterns in the lower extremities of sheep during locomotion.^29,56,57^ Instrumented scaffolds demonstrated high sensitivity, detecting changes of 29% and 8% in the principal compressive strain when a non-dynamized and dynamized animal increased their walking speed, respectively. Burr *et al.* noted a similar trend while measuring human tibial strain during vigorous activity.^58^ The principal compressive strain increased in magnitude by 10% when the subject went from jogging to sprinting. The magnitude of change in the principal compressive strains when animals changed speeds could be explained by the healing progression of two animals and the amount of bone in the defect space. *In vivo* monitoring of compressive forces and changes in principal strain noted a progressive decrease in scaffold deformation and loads during bone regeneration. During healing, a change in strain transfer between the scaffold and bone would be expected. Szivek *et al.* previously described a strain transfer change when utilizing sensate polymer scaffolds as part of large cartilage defect regeneration studies.^12^ A finite element model designed to replicate *in vivo* strain transfer between bone and the implanted scaffolds noted a relative decrease in scaffold strain as bone remodeling progressed.^59^

While the use of mechano-sensitive sensors such as strain gauges demonstrates a possible use for monitoring healing of bone defects, their limitations should be taken into consideration to achieve bone healing insights. The incorporation of electronic systems in a biological environment necessitates appropriate and reliable data acquisition methods. The use of transcutaneous wires is not ideal for long-term data acquisition due to the likelihood of infection. Additionally, water infiltration and mechanical failures of wires in this study prevented *ex vivo* evaluation of the system. Better encapsulation methods should be developed to prevent fluid infiltration. In this study, four animals demonstrated the feasibility of adopting wireless, battery-free telemetry units for continuous monitoring of bone health parameters, as previously demonstrated when monitoring cartilage defects (supplementary material, Fig. 6).^12,13,35,60^ Our lab recently highlighted the development and use of reliable, thin, and non-bulky wireless, battery-free, flexible systems called osseosurface electronics.^61^ Osseosurface devices provide a platform for monitoring bone health parameters in the long-term that does not affect physiological behavior. Parylene-C encapsulation of these osseosurface devices provides precise bone strain measurements *in vivo* for up to 11 months.^62^ These osseosurface electronics can be incorporated with biosymbiotic wearable technology for continuous wireless power transfer and communication for real-time continuous data acquisition.^62,63^ The adoption of such systems can provide better insights into long-term healing of critical size bone defects through reliable and precise strain monitoring.

In summary, our results demonstrate that dynamization and hardware removal used to treat critical size bone defects utilizing 3D printed biomimetic ceramic-coated PBT scaffolds improved the union rate and strengthen the characteristics of regenerated bone compared to a non-dynamized group. Additionally, we demonstrate the use of mechano-sensitive sensors for monitoring scaffold deformations as a viable method for real-time monitoring of bone regeneration and healing. Additional research is warranted using updated wireless, battery-free systems for the long-term assessment of bone healing.

## METHODS

### Scaffold preparation

**FIG. 7.**
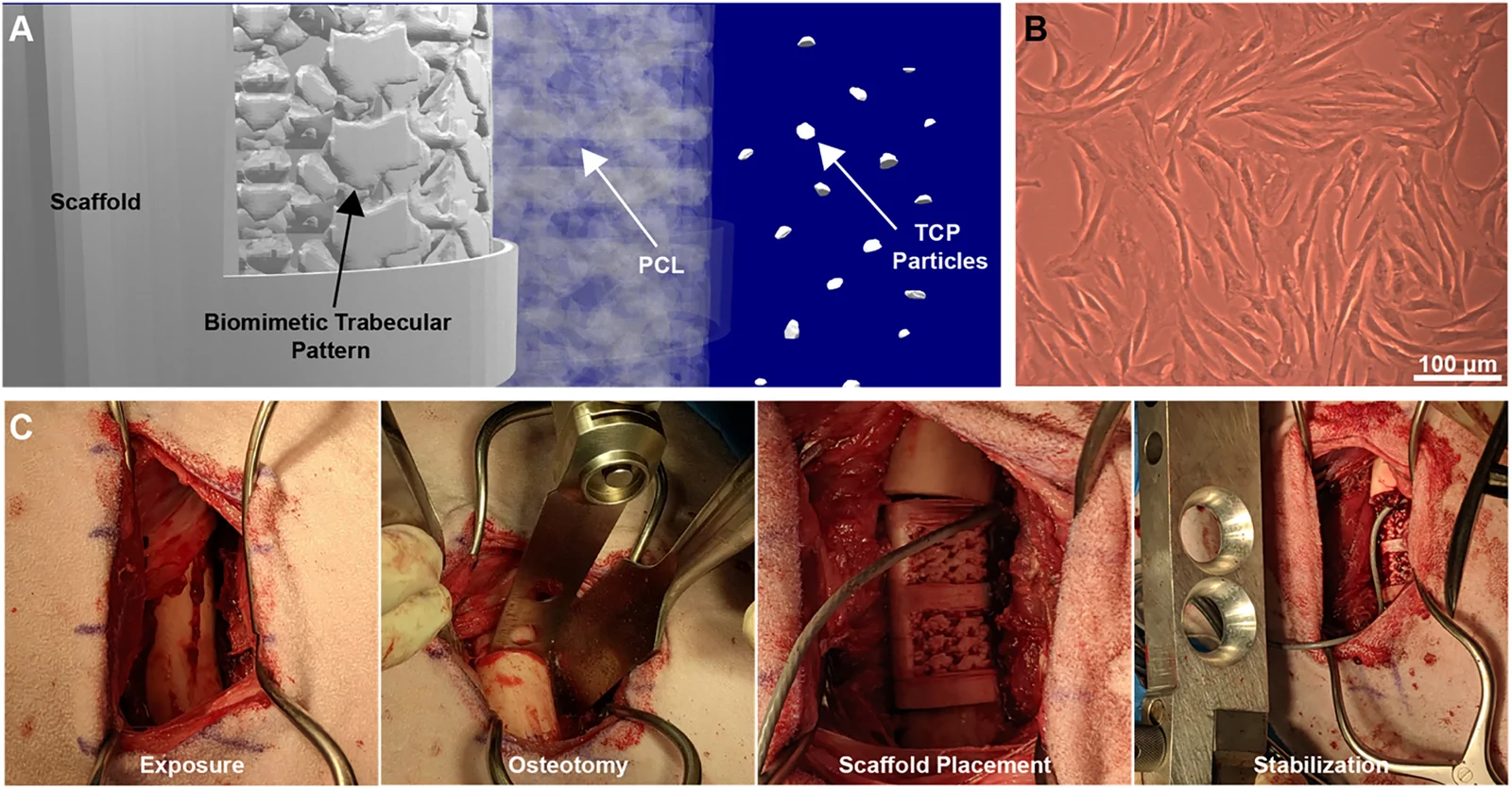
Scaffold preparation. (a) 3D rending of scaffold layers containing PCL and TCP particles as an osteoconductive layer. (b) Microscopy image of cultured adipose derived stem cells. (c) Scaffold implantation procedure showing the exposure of the femur, performing a cortical osteotomy, placing the scaffold, and stabilizing the defect with an intramedullary nail.

Cylindrical scaffolds measuring 4.2 cm in length and 2.2 cm in diameter were printed with PBT (Sabic Polymershapes, Huntersville, NC) in a Stratasys 1650 Fused Deposition Modeler (FDM, Stratasys, Eden Prarie, MN). Scaffolds had an inner biomimetic porous structure derived from inverting *μ*CT scans of sheep femoral trabecular cores scanned at a 12 *μ*m resolution.^14,15^ Inverting the *μ*CT scan creates a scaffold with material occupying the marrow space while leaving the bone space empty to facilitate bone regeneration with the original structure. The inner structure was surrounded by an outer shell with 2 solid struts to facilitate strain gauge attachment. Three 1000Ω uniaxial strain gauges in a rosette pattern were glued to the lateral side of the scaffold using medical grade epoxy (EP42HT, MasterBond, Hackensack, NJ). The custom rosette pattern was designed with strain components in the axial (270°), shear (135°), and transverse (0°) axes [supplementary material, Fig. 3(a)]. Strain gauges were wired using 32-gauge multiconductor cables connected to a 6-pin circular connector (Cooner Wire, Chatsworth, CA). The connector-wire interface and the connector end were reinforced and waterproofed using heat shrink and epoxy. Scaffolds were then coated in β-TCP by submerging the scaffold into a mixture of 4 *μ*m sized β-TCP particles and Polycaprolactone (PCL) in a ultrasonic cleaner and vacuumed to allow the β-TCP particles to infiltrate the pores [Fig. 7(a)].^9^ Scaffolds were then heated to 75 °C and rinsed in de-ionized water.

Four animals were implanted with a four-channel digital radio transmitter (Microstrain, Williston, VT).^12^ Scaffolds were prepared and instrumentalized as previously described. The 6-pin circular connector was made to interface with the radio transmitter before reinforcing and waterproofing the connection with polyurethane (M-Line, Raleight, NC) and epoxy. The radio transmitter was made to couple with an external radio receiver connected to an external computer.

The rosette strain gauges on the scaffolds were calibrated using nondestructive compressive mechanical testing with servo hydraulic Mechanical Testing System (MTS, Minneapolis, MN) to generate a load-strain curve. The scaffolds were loaded 6 times using a ramp function with an ascending rate of 49 N s^−1^ to peak loads of 490 N. The elastic deformation region of the compression test was used to calculate scaffold stiffness [supplementary material, Fig. 3(b)]. Load–strain curves were calculated for all three strain gauges within the rosette strain gauges for calibration. Standard strain transformation equations were used to analyze change in direction and magnitude of the compressive and tensile principal strains and the maximum shear strain during compressive loading. Equations were adapted to the strain gauge orientation of the custom rosette set up, as shown in the supplementary material, Fig. 3(a). The characterization of scaffold mechanical properties including Poisson's ratio and anisotropy ratio was also calculated [supplementary material, Figs. 3(c) and 3(d)]. Scaffolds were then cleaned in de-ionized water, sterilized using ethylene-oxide, and seeded with stem cells prior to surgical placement.

### Surgical procedures

#### Autologous adipose derived stem cell isolation and seeding

All animal experiments were approved by the IACUC. Two weeks before implantation, adipose derived stem cells were obtained from the tail fat pad of the sheep and processed using a published procedure.^27^ The tissue and extracellular matrix were mechanically broken and subsequently enzymatically digested in collagenase to release the cells. Cells were expanded in Dulbecco's modified eagle medium (DMEM, Gibco) supplemented with 10% fetal bovine serum and 1% penicillin/streptomycin under 37 °C and 5% CO_2_ [Fig. 7(b)]. One day before implantation, 1.5 × 10^6^ cells were counted and seeded onto the surface of scaffold and allowed to attach overnight.

To test the distribution of cells within the scaffold, gold nanoparticle labeled cells were seeded on scaffolds and imaged using *μ*CT. Briefly, cells were cultured in DMEM media containing 20 nm diameter gold nanoparticles (Sigma-Aldrich, Burlington, MA) at a volumetric ratio of 2:3 overnight. Gold nanoparticle infused cells were trypsinized, and 1.5 × 10^6^ cells were seeded on the scaffold surface. Cells were left to attach for 1 day to replicate the implantation conditions. Seeded scaffolds were rinsed three times with PBS, fixed with 4% paraformaldehyde (Sigma-Aldrich, Burlington, MA) for 90 min, and again rinsed three times with PBS. Imaging of the gold nanoparticle infused cells was performed using μCT (Inveon *μ*CT scanner, Siemens, Malvern, PA) and visualized on DragonFly v. 2022 (Object Research Systems) to observe cell distribution within the scaffold.

#### Creation and treatment of a segmental femoral defect

A large segmental mid-diaphyseal femoral defect was created in sheep, and a scaffold was placed in the defect using a published procedure^28^ [Fig. 1(c)]. The procedure was performed on 19 sheep weighing between 49 and 81 kg. Briefly, an 8 cm lateral thigh incision was made. The interval between the quadriceps and hamstrings was developed to expose the femur. A 4.2 cm defect was created using a custom cutting guide. A scaffold was placed into the defect. The scaffold and femur were stabilized using a custom 9 mm diameter intramedullary nail with proximal and distal interlocking screws. The control group did not receive a scaffold but did have a 4.2 cm defect stabilized with an intramedullary nail. The multichannel cable connected to the scaffold was guided subcutaneously along the back of the animal and sutured in place just proximal to the shoulder blades. Proper placement of the scaffold and stabilizing hardware was verified with radiographs.

#### Dynamization and hardware removal

After 6-months, 10 sheep with scaffolds underwent dynamization by removal of the proximal interlocking screw [supplementary material, Fig. 2(a)]. Radiographs were taken to localize the proximal interlocking screw, which was removed through a 3 cm incision. 3 months post-dynamization, 4 sheep underwent the removal of the remaining stabilizing hardware [supplementary material, Fig. 2(b)]. Control sheep were held for 6 months post-implantation while non-dynamized and dynamized sheep were held for at least 9 months post-implantation. Sheep in the hardware removal group were kept for up to 1 year following the removal of all hardware. An illustration describing each experimental group and their respective timeframes is shown in the supplementary material, Fig. 1.

### *In vivo* testing and imaging

Monthly radiographs were taken following the creation of the segmental defect using a Stalo ultrahigh-definition x-ray acquisition station (Universal Imaging, Stalo Series Canon). Images were taken in the lateral and anterior–posterior planes to document the bone growth across the defect site.

For animals undergoing dynamization or hardware removal, wires were exposed 4 days prior to secondary surgeries. Animals that did not undergo a secondary surgery had wire exposure at 9 months with wires left exposed for up to 2 months.

Multiconductor cables were connected to a multichannel data acquisition system (System 800, MicroMeasurements) and running at 100 Hz to record *in vivo* strain data. Data were recorded, while animals were walked on a Trotter treadmill (United Medical Company, Mills, MA) at a minimum speed of 2.6 km h^−1^ and during *ad lib* walking. Animals walked for up to 20 min. The period of lowest strain during the swing phase was used as zero strain, assuming the limb would be under the least amount of load in agreement with Lanyon.^29^ The same equations shown in the supplementary material, Fig. 3(a) were used to evaluate the change in orientation and magnitude of the principal strains during treadmill walking. Calibration curves obtained from each scaffold before surgery were used to assess loads passing through the scaffold. Limb loads were calculated from the total load change in each gait cycle.

### Radiography and computed tomography

Femora were explanted, radiographed, and cleaned of soft tissue before removing transcortical screws and the intramedullary nail. Bones were maintained in PBS-soaked gauze to prevent dehydration and scanned using an Inveon μCT scanner (Siemens, Malvern, PA) with a layer distance of 105 *μ*m. The imaged segment was 8.5 cm, with a 4.2 cm of the defect site plus 2 cm above and below the segment. DICOM images were retrieved and analyzed using DragonFly v. 2022 (Object Research Systems). Parameters measured included total cortical bone (cm^3^), bone ingrowth (%), cortical bone porosity (%), and the polar moment of inertia (pMOI) (mm^4^). Bone ingrowth was calculated as the volume of bone inside the scaffold area relative to the total bone volume in the defect space. Cortical bone porosity was calculated by dividing the cortical bone porosity volume by the total cortical bone outside the scaffold area (supplementary material, Fig. 4). Bone distribution analysis was conducted by analyzing the pMOI for individual transverse slices along the defect site.

### Mechanical testing

After *μ*CT scans, bones underwent nondestructive mechanical loading using a MTS. Bones were vertically potted in fixture using a low-melting point alloy (Cerrobend, Scottsdale Tool, AZ). Bones were tested 6 times in axial compression, anterior cantilever bending, and posterior cantilever bending. During compression, femora were placed parallel to the loading pin, applying a direct load to the femoral head. During cantilever bending, femora were placed perpendicular to the loading pin, applying a bending load to either the anterior or posterior face of the femur. Whole bone stiffness was calculated from the linear portion of the *Load v. Deflection* curve.

### Histology

Following imaging and mechanical testing, the explanted bones were fixed in 10% Neutral Buffered Formalin, dehydrated in a series of ethanol (70%–100%), and embedded in a polymethylmethacrylate resin (PMMA, Polysciences Inc, Warrington PA). A cross-section was cut through the middle of the scaffold, ground using 120–600 grit silicone carbide paper, and polished. The slides were stained using Villanueva Osteochrome Bone Stain (Polysciences Inc, Warrington, PA) and imaged using a motorized microscope at 10 times magnification (LeicaDMI600, Leica Microsystems Inc, Deerfield, IL). Brightfield images were analyzed using the Bioquant OSTEO software, which follows the guidelines set by the American Society for Bone and Mineral Research. Polarized light imaging was performed to quantify the birefringent collagen, which was analyzed using ImageJ. Histological measurements included cortical bone area (mm^2^), endocortical bone area (mm^2^), birefringent area fraction (%), and birefringent intensity (mean gray scale).

### Statistical analysis

All data analysis was done using Graphpad (Graphpad Software, CA). *In vivo* bone strain data are shown as strain and load change across the scaffolds for individual animals. Bones that did not complete the entire mechanical testing protocol secondary to nonunion or failure during testing were included in the statistical analysis and represented as a stiffness of 0 N mm^−1^. A One-Way ANOVA was used to compare across individual groups. Statistical significance was observed when ^*^p < 0.05.

## SUPPLEMENTARY MATERIAL

See the supplementary material for additional details on methods and data.

## Data Availability

The data that support the findings of this study are available from the corresponding author upon reasonable request.
